# Public Health Education Using Pop Culture and Media

**DOI:** 10.3389/fpubh.2014.00231

**Published:** 2014-11-10

**Authors:** Brandon Brown

**Affiliations:** ^1^Program in Public Health, Department of Population Health and Disease Prevention, University of California, Irvine, CA, USA

**Keywords:** education, public health, media, popular culture, videos, global health

## General Public Health Knowledge-Action Needed

Public health emphasizes disease prevention, health promotion, and treatment at the population level. General knowledge about public health is lacking, despite the international growth of public health programs. The most recent public health opinion poll identified in the United States (US) was completed over 20 years ago in 1996 by Louis Harris and Associates. The random digit-dialing project revealed that a majority of US respondents viewed public health services, such as preventing the spread of infectious diseases and implementing healthy lifestyle programs, as very important (1). Still, the definition of public health remains ambiguous or unknown (1–3). When over 1000 adults were asked to define public health, 36% said it is the well-being of the public, 12% did not know, 11% said it is a government-provided health care system, and 9% said it was a government-provided system for the needy and elderly (3). Less than 4% of respondents identified public health with disease prevention or health promotion (3). Though this poll took place some time ago, if the trend continues, most Americans are likely still unable to define public health. This may not all be due to low-public health knowledge, but perhaps some due to low-health literacy. According to the US National Adult Literacy Survey, nearly half of the US adult population has inadequate reading or computing knowledge, and estimates show one-third of adults need assistance with interpreting health terminology (4, 5).

## Precedent for Use of Technology in Learning

We are moving more into utilizing technology for teaching and communication. In fact, technology is difficult to avoid, and many students prefer to use a laptop or tablet rather than a pen and paper in class. In a world where new technology emerges on a daily basis, more and more Universities are offering online and hybrid courses that utilize these advances. From 2007 to 2013, undergraduate student enrollment in at least one distance-learning or online course increased from 20 to 32% (6–8). Public health schools and programs are no exception to this trend. As of spring 2013, the Council on Education for Public Health (CEPH) accredited 87 schools and programs that offer online public health degrees (9). Some of these courses are conducted entirely online, while hybrid courses combine elements of both online and in-person learning. Still, most University courses follow the traditional in-person lecture format, including very few components of online learning (10).

Online courses may be just as good or better than in person lectures. Over three-quarters (77%) of senior academic officers rated online education learning outcomes as good or better than in-person instruction (7). The hybrid learning approach led to better academic performance among graduate students than the traditional approach as well (10). With respect to courses that are conducted entirely online, research reveals wide variation in students’ level of participation, with more students viewing video lectures for quantitative courses compared to non-quantitative courses (11). One study found that including case studies in online courses can produce the student engagement and active learning typically associated with traditional, in-person teaching (8). Furthermore, students report that the flexibility of online courses facilitates a deeper understanding of the material (8). Students are more tech savvy than ever, and online and hybrid courses exploit this strength. Some experts in university education argue that the best way a student can learn is by doing. The rote memorization method, which may be useful in preparing for a written exam, is not a viable method for students to learn the skills they need to excel in the real world. This issue is particularly relevant for public health, a topic that requires interactive learning and real world application. Creation of public health educational content by students including pop culture is one potential strategy to achieve interactive learning and develop leadership skills, though this method has not been previously used. The diffusion of innovation theory would potentially assist in further replication of video creation (12).

## Creation of Public Health Videos to Educate Peers

Undergraduate students in upper-division public health courses at UC Irvine were offered an extra credit opportunity to develop videos defining public health and global health using pop culture as a tool. While use of pop culture by students to educate on topics is relatively unused in public health, it has been utilized broadly, including airline safety videos. Delta Airlines is on their sixth in-flight safety video, which uses humor to deliver low-interest safety messages (13). The overall goal of our video project was to help communicate public health to individuals, both in the UC Irvine community and beyond. This include student learning by those creating the videos, peer learning by students viewing the videos, and community learning for others viewing the videos both inside and outside of UC Irvine. This was deemed important given the relatively low knowledge of the public health role outside of public health departments due to the population based nature of our work.

The rubric was simple. For an extra credit assignment, students were asked to define public health or global health for their peers. They were asked to create a 2–5 min video, which must have a health message and using pop culture to reach the general public. Creation of narratives and storytelling was recommended according to Fishers narrative theory (14). Submissions were rank ordered by an external panel of judges with expertise in public health, who also gave comments and nominated videos for an award (best storyline, most educational, most hilarious). They were informed that many people in the general population do not know what public health is, and that we want to reach this group. They could choose any theme, health topic, song they wanted as long as the video educated on public health.

**Figure 1:**
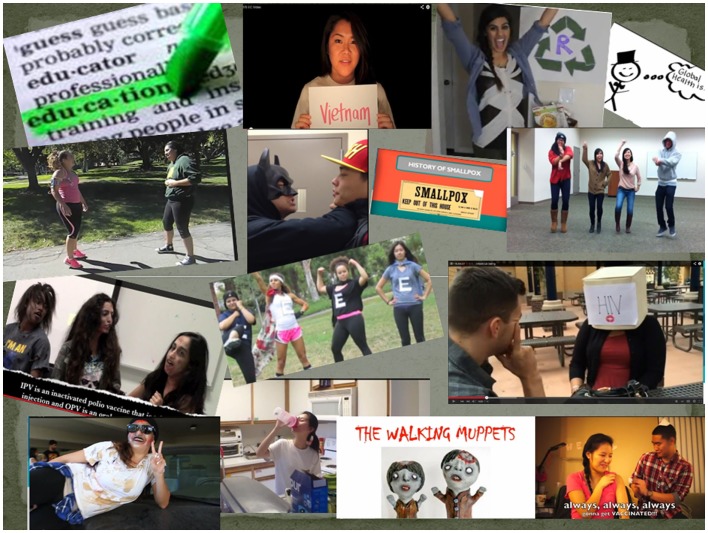
**A collection of pictures from student led public health education videos**.

The project was a major success, with 35 videos created by individuals and groups of students from June 2012 to August 2014, and several appearing on public health websites. All videos included student appearances, and some included students themselves singing (*N* = 14) and dancing (*N* = 7), and with popular background songs (*N* = 19) as they provided their health message (Figure 1). Topics varied greatly, from HIV and disaster preparedness to vaccination and mental health awareness (Table S1 in Supplementary Material). Across 3 years of the video project, students used popular themes including dating diseases and pop culture from Walking Dead, the Rocky soundtrack, Michael Jackson’s ABCs, Superheroes, and Power Rangers. Very high-quality videography was done using standard telephones with recording features.

When given the opportunity, students can utilize media (songs, movies, and plays) and employ innovative methods including use of pop culture to successfully educate their peers about issues of public health significance. These peer driven methods are more likely to reach and educate their peers and the general community outside of UC Irvine, and to increase knowledge about public and global health. We must continue to use all the tools in our toolkit to educate on public health. Online media, student led peer education, and pop culture are a few of these tools.

## Recent Success and Next Steps

Our innovative use of pop culture in educative videos has resulted in initial success inside and outside the classroom. In total, the YouTube hits on these videos ranged from 29 to 841 views, which is a wide range. Views may have been due to the pop culture component of the message, or larger networks of the student videographers. With 849 views, we hope that the public health messages were passed on to students and the general public who are less familiar or less interested in public health in general. Judges recognized videos as being most entertaining, best quality, strong public health message, best script, and most creative, which yielded some data on project quality. The videos received attention in the APHA newsletter, The Nations Health, in an article titled California students use videos to promote health: students make global public health shine on the small screen (15). We also presented details of our video assignment for other schools to initiate student peer public health education videos at the 2013 APHA Undergraduate Summit titled Creative Learning by Teaching Using Media-Undergraduate Public Health Students in Action. On campus, the video project is well known and highlighted on our public health GHREAT Initiative website (http://ghreat.uci.edu/).

As educators of public health, we can use these videos and the pop culture included in them as a framework to educate the public on topics, which are of less interest. We showed this recently in a paper focused on rabies and using zombies as a comparator, and the Nations Health published a piece on delivering health messages with comics (16, 17). A call to action to all schools and programs in public health is in order to create similar videos in their courses to teach both students and the general population about public health topics. An important component of gauging the success of these videos would be with an evaluation of public health knowledge, done in several ways. At the classroom level, we can compare learning objectives and student evaluations of courses prior and following the addition of the public health videos. In the general public, we can initiate a survey on changes in attitudes, knowledge, and behavior. As a starting point, we can ask questions including the definition of public health, current public health concerns, and interest in public health. This survey can be done before and after viewing the videos to show change in knowledge and public health interest from viewing public health educational videos. Additional questions following the video would include the viewer perspective on the purpose of the video, use of pop culture and humor to increase awareness, clarity of the public health message, and the potential video impact on public health education for their peers. We can also request a response for ways of improving the videos to further enhance health communication.

As a faculty member who uses these videos as a component of teaching in my courses, I will be moving from requesting videos for extra credit to making the group videos a requirement in my courses, and we will expand to creating the videos in my public health ethics course. Similarly, my colleagues have worked to nurture the creation of these videos in public health nutrition and global health courses. We can link all of these on a national website by course and topic so that we have a compendium of videos to refer to when appropriate. The general public can rate the videos and the winners can be recommended for national campaigns. It is time public health gained the national and international notoriety it deserves. We now have an additional tool to achieve this goal.

## Author Contributions

Brandon Brown is the sole author of this manuscript.

## Conflict of Interest Statement

The author declares that the research was conducted in the absence of any commercial or financial relationships that could be construed as a potential conflict of interest.

## Supplementary Material

The Supplementary Material for this article can be found online at http://www.frontiersin.org/Journal/10.3389/fpubh.2014.00231/full
