# Supplementary material for: Public Health Education Using Pop Culture and Media
Source: Front Public Health. 2014 Nov 10;2:231. doi: 10.3389/fpubh.2014.00231 (PMC4226141; doi:10.3389/fpubh.2014.00231)
Supplement: Supplementary file 1 [file Table_1.PDF]

Supplementary Table 1. Video themes and public health topics created by public health students

Year	Video Themes	Topics
2012	The Eye of the Anteater-Rocky Fight Song, Healthy Lifestyle, Public Health Cleaning service, What is Global Health, Public Health News Report, Global Health Cartoons, Call to Action, Batman, Big 3 Diseases, Public Health Style-Gangham Style	vaccination, preventing diseases, HIV, TB, malaria, eating right, exercise, diabetes, heart attack, nutrition, smoking, drinking alcohol and drug use, national borders, personal definitions of global health, health care, mental health awareness, population control, handwashing, flu, obesity
2013	HIV, The Office (UCI edition), Save the planet-become a Global Ranger, Healthy Lifestyle, Global Health, Public Health Course Infomercial, Global Health Today - KCAL 170, Give Love-Give Health, The Big 3 Diseases Eradicators-superheroes, Zombie Apocalypse, ABC's of HIV, HIV transmission	rabies, public health awareness, education, malaria, HIV, TB, clean environment, eating right, covering your mouth when you cough, the big 3 diseases, malnutrition, identify global health issues and teach your peers, public health issues around the world, environmental health, obesity, unprotected sex, hiv testing, identify and eliminate health problems, emergency preparedness
2014	Infectious Dating, Zombie Jeopardy, Shaken Baby Syndrome, The Walking Muppets-Walking Dead, Healthy Diet, Smallpox Eradication, The Rapping Dead, Sharing and Caring, Obesity, Public Health Speed Dating	safe dating, public health awareness, HIV, TB, malaria, baby handling, awareness and prevention of rabies, healthy life style, exercise, smallpox history, vaccination, sharing needles and HIV exposure, characteristics of different infectious diseases
